# Do expressive writing interventions have positive effects on Koreans?: a meta-analysis

**DOI:** 10.3389/fpsyt.2023.1204053

**Published:** 2023-11-23

**Authors:** Yeseul Lee, Dongil Kim, Jung-Eun Lim

**Affiliations:** Department of Education, Seoul National University, Seoul, Republic of Korea

**Keywords:** expressive writing, meta-analysis, writing therapy, emotional exposure, self-disclosure, effect size

## Abstract

**Introduction:**

Expressive Writing (EW) is an intervention that focuses on individuals’ writing down their thoughts and feelings about trauma or stressful events. Meta-analyses on EW studies have confirmed that EW has a positive effect. However, the heterogeneity of studies is high, so many studies have investigated boundary conditions and moderators. One of these moderators is the cultural difference in emotional suppression. Since EW focuses on the expression of suppressed thoughts and emotions, its effect might be slightly different for people in Asian cultures who show a high tendency to suppress their emotions. This study attempted to confirm the effect size of the EW interventions in Korea and examine whether these studies have different effect size from those based on Western cultures.

**Method:**

A total of 29 studies published in Korea until 2021 were analyzed. The effect size was calculated using the “dmetar,” “meta,” and “metafor” packages of the statistical program R 4.0.4.

**Results:**

The results were as follows. First, the effect size of EW intervention was 0.16, and we found that studies in the Korean context showed no significant difference from studies based on western meta-analysis. Second, the moderating variables that influenced the EW intervention were the writing type, the number of sessions, the time per session, and the measurement time.

**Discussion:**

The results of this study suggest that EW interventions benefit Koreans. And it is at least harmless and has a positive effect considering the efficiency and conciseness of interventions. Furthermore, the finding shows that EW interventions can be helpful even in the general population without apparent psychological problems. By considering moderators, we could structure more effective form of EW interventions for Koreans.

## 1 Introduction

Expressive Writing (EW) interventions focus on facilitating participants to freely write down thoughts and feelings about traumatic or stressful events (1). Since Pennebaker and Beall (2) published a study in which EW is found to be beneficial for physical health, EW interventions have been continuously explored. Pennebaker and Beall asked participants to write down their deep thoughts and feelings about trauma or stress continuously for 3–4 days and for 15–20 min a day (2). When writing, spelling, or grammatical accuracy were not considered important; it is important to let participants keep writing without a pause (3). As research on EW interventions continues, the terms that refer to these interventions differ slightly among studies. For example, terms such as expressive writing (4), experimental disclosure (2), written emotional disclosure (5), and writing therapy (6) have been used. Despite these differences, the common feature of all studies is that they were based on Pennebaker and Beall’s study on EW (1). Contrary to the original intervention in which participants were asked to write down whatever came to their minds, structured EW was developed to guide the content to be written down during each session in detail. For example, Guastella and Dadds developed a three-step structured EW process: “exposure - devaluation - benefit finding” (7).

Disclosure of participants’ difficulties is a basic element in counseling and psychotherapy (8). Counseling in EW interventions has several advantages. It is a treatment that clients can try on their own without the direct intervention of counselors. Further, EW intervention is low-cost, low-risk, and timesaving (9, 10) and is relatively safe because it is not invasive (11, 12). EW interventions are exploratory and work indirectly, so it can be useful for resistive or defensive clients (13). Above all, since EW intervention does not require direct face-to-face interactions, it can be presented as a task during counseling sessions (14) and in an online scenario (15, 16). It can be a very useful intervention for those for whom therapy is not available option for reasons such as expense, lack of access to affirmative providers, or internalized stigma (17).

Many studies have proven the benefit of EW interventions for various participants. In early studies, EW was primarily targeted toward college students, and it was found to be beneficial for physical and mental health, including blood pressure and negative emotions. As studies progressed, the participants gradually expanded and diversified, including the unemployed (18), prison inmates, (19) and sexual minorities (12). EW interventions have also been extended to participants suffering from certain diseases and disorders, such as post-traumatic stress disorder (20–23), arthritis (24), cancer (25–28), asthma (29, 30), and eating disorders (31, 32).

The benefits of EW intervention have been investigated in three categories: psychological health, physical health, and general functioning. Studies examine physical health changes by assessing individuals’ blood pressure, heart rate, and cortisol levels (21, 33, 34). Psychological health changes were mainly studied in relation about the reduction of negative emotions such as depression and anxiety (18, 22, 35–41). General functioning changes were investigated through an assessment of the rate of absenteeism (42), academic performance (34, 43, 44), and working memory (45).

Not all these steadily accumulating EW studies have resulted in beneficial outcomes. Several studies concluded that there was no difference between the EW intervention group and the control group (30, 46, 47). Additionally, some studies show that EW intervention has produced negative results, such as increased hospital visits (48, 49). These conflicting results have sparked controversy over the effectiveness of EW interventions (48, 50, 51).

Therefore, a meta-analysis was attempted to synthesize studies on the writing intervention and determine the effect size (2, 5, 52). In a study by Smyth (52), the first meta-analysis of EW interventions, the effect size was 0.47, which means that the interventions had a moderate effect. However, a total of 13 studies were included in the analysis at the time, and it is difficult to generalize about these studies because a fixed effects model was used. Another limitation is that most studies were conducted on college students or individuals who did not have any major psychological problems. In a study by Frisina et al. which covered these limitations and analyzed only the clinical population, the effect size was 0.19 (5). In Frattaroli’s study, which applied a random effect model, including both published and unpublished studies, the effect size was 0.15 (2).

Recently, meta-analyses have been attempted to prove the effect of EW interventions on specific areas by narrowing the focus of research. For example, a study by Qian et al. examined EW interventions among pregnant women (53). Other studies target individuals with posttraumatic stress (6) and caregivers (54). For caregivers, EW reduces trauma symptoms and improves psychological health, but the effect was not significant on depression, anxiety, physical symptoms, quality of life, and caregiver burden (54). In the case of the post-traumatic stress group, EW intervention influenced post-traumatic stress disorder, but the effect was not significant on anxiety and stress symptoms (6).

Combining these research results, we could see that EW interventions had a statistically significant positive effect, albeit at a small level. However, the heterogeneity of each study is high (2). Despite growing evidence for this intervention, its boundary conditions are still unclear. Research findings suggesting that EW may be more effective for specific participants and situations have led to an interest in the groups which might benefit from it (4, 55, 56).

Considering that the core of EW is about expressing emotions, researchers have been interested in moderators, such as expressiveness and emotional ambivalence (25, 57). In particular, Smyth found in a study that the male group benefited more than the female group (52), suggesting that individuals with high emotional suppression would benefit more. In this context, a review of race and culture as moderators of emotional suppression has begun. As shown by many studies, individuals from Asian cultures do not tend to express their negative emotions and suppress them more than individuals from Western cultures (58–61). Owing to the nature of their culture which values harmony and peace, Asians are reluctant to express or conflict directly (62, 63) and tend to consider negative emotions as a sign of their vulnerability and weakness (64). This is also true of Koreans. In Korean culture, emotional expression is inappropriate—something to be controlled inwardly and not to be expressed (65).

Considering these points, one can assume that EW interventions that facilitate the exploration and exposure of normally suppressed thoughts or emotions may be more effective for Asians than for Caucasians (66). In the study of Lu and Stanton on Caucasians and Asians (56), the effect of EW was found to be greater on Asians than on Caucasians, especially in the improvement of physical symptoms. Frattaroli also predicted that EW intervention would be more effective for individuals from Eastern cultures (2), who tend to suppress emotions. However, about 7% of the total study participants were Asian, and the results of the study did not statistically support its hypothesis.

A meta-analysis conducted on the effectiveness of expressive writing targeting the Asian population found the effect size to be very low at 0.05 (67). Indeed, individual studies examining the effects of expressive writing interventions on Asian populations show inconsistent results in their effects. For instance, a meta-analysis on the effect size of expressive writing intervention on anxiety reduction among Chinese breast cancer patients yielded a significantly large effect size of 1.22 (68). However, a study targeting Korean breast cancer patients found no significant effect on anxiety reduction (69). This suggests that even within the same Asian cultural sphere, there may be varying patterns of effectiveness depending on the country, such as Korea, China, or Japan. This is because, within the Asian cultural sphere itself, there are differences in emotional expression, as seen in Korea, China, and Japan (70). For example, Japanese individuals tend to express their emotions less than Koreans, and they may even feel a greater need to suppress their expressions (71). Additionally, a study on the emotional differences of ‘shyness’ and ‘intimacy’ among Koreans, Chinese, and Japanese found that compared to the other two countries, Koreans scored lower on shyness and higher on intimacy (72). Thus, even within the same Asian cultural sphere, there can be significant cultural diversity, and above all, due to differences in societal norms regarding the perception and expression of emotions, as well as emotional suppression, the effects of expressive writing may manifest in slightly different patterns.

Therefore, this study aimed to examine the effect size of EW interventions conducted in the native language on Koreans, who belong to the Asian culture. Specifically, this study will examine the overall effect size of EW interventions conducted in Korea and examine the moderators influencing the effect.

The research questions of this study are as follows.

(1) What is the global effect size of EW interventions included in the meta-analysis?
(2) What are the moderators that affect the effect size of EW intervention?

## 2 Method

### 2.1 Literature search

**FIGURE 1:**
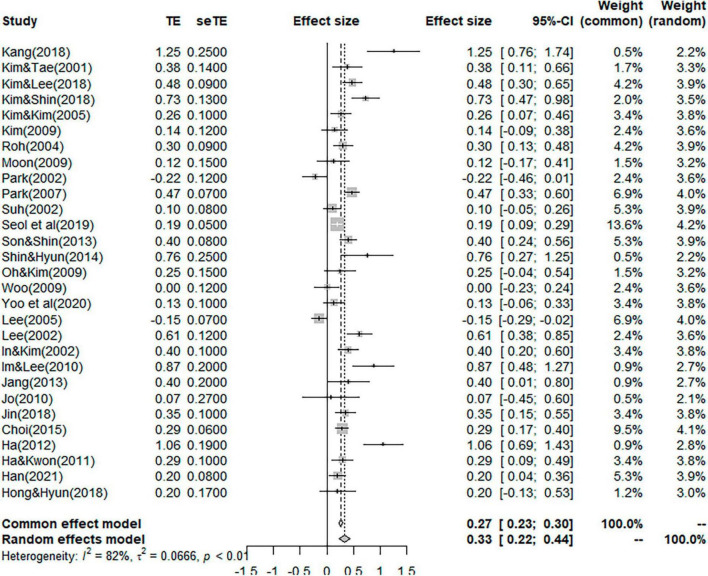
Funnel plot test.

For the selection of individual studies for the analysis in this study, we followed the PRISMA criteria proposed by Moher et al. (73), as depicted in Figure 1. The specific methodology is outlined as follows. To select relevant studies, an online computerized search was conducted among three major databases of academic materials in Korea: The National Assembly Library, RISS (Research Information Sharing Service), and KISS (Korean Studies Information Service System). During the search, we set *writing* as a necessary term to be part of the title, abstract, and keywords from each study, with a combination of the following terms: *expressive, expression, therapy, self-disclosure, emotional, counseling*, and *program.* We included dissertations as well as journal articles. While searching, we did not put a limit on the starting point of the publication date, so articles published until December 2021 were included.

### 2.2 Criteria and procedure for selection

The inclusion and exclusion criteria were as follows. First, the study of EW intervention had to be based on Pennebaker and Beall (1). Writings about objective facts, such as newspaper articles, were excluded. Studies that included other therapeutic elements (e.g., coloring, mindfulness meditation) besides EW interventions were also excluded to investigate the effect of EW alone. Second, experimental or quasi-experimental studies in which a control group and an experimental group existed were included, where the groups could be compared with a “pre-post-group design.” Third, a study that presented statistical information sufficient for the calculation of an effect size was included.

First, according to the criteria of PRISMA (74), 1022 studies were selected after the duplicates were removed. Thirteen dissertations were published as journal articles and were analyzed. Second, we screened materials by titles and abstracts and excluded 909 studies irrelevant to EW interventions. Then, 113 studies were reviewed. Four studies whose full text could not be accessed were excluded and 80 studies were excluded for not being experimental studies (case studies, theoretical studies, and qualitative studies) (*n* = 52), not reporting the required statistics (*n* = 7), and for investigating counseling programs including other interventions besides EW (drawing, meditation, etc.) (*n* = 21). Finally, 29 studies were selected for analysis.

### 2.3 Coding procedures

#### 2.3.1 Coding system for quality

Study quality may influence the effect size, and quality rating was done before meta-analysis. For quality rating, two reviewers independently rated the quality of every study according to the Joanna Briggs Institute (JBI) Appraisal Checklist (75). The JBI quality appraisal tool for randomized controlled trials consists of the following 13 questions: randomization assignment; concealed groups allocation; pre-homogeneity of each group; subject blind; experimenters’ blind to treatment assignment; measurer blinding; identical conditions other than experimental treatment; appropriate follow up; participants analyzed in the groups to which they were randomized; homogeneity of outcome measures in each group; outcomes measured in a reliable way; appropriate statistical analysis, appropriate trial design. The quality of each study was evaluated by two researchers independently. For “yes” to a question, 1 point was given. For “no/unclear” to a question, 0 points were given. The result was analyzed by adjusting the consensus and disagreement between the reviewers through a researcher meeting. In consideration of previous studies (76), the studies for meta-analysis were selected if the number of “yes” items was more than half (7 points or more). All 29 studies scored 7 points of higher. Therefore, a total of 29 studies were ultimately included in the analysis.

#### 2.3.2 Coding system for meta-analysis

The frame adopted for coding analysis used in this study is shown in Table 1. The categories of variables were prepared by referring to previous studies (2, 5, 52) after discussion by two doctoral students. Afterward, the contents were reviewed by one professor majoring in counseling; thereafter, the framework was revised and supplemented. In coding the pre-post score, we aimed to standardize the direction of positive and negative variables. Specifically, we treated the decrease in negative variables and the increase in positive variables in the same direction. In this study, all variables were treated in the same direction through reverse coding.

**TABLE 1:** Coding table.

Category		Sub-category
Basic information		Number, published year, author, title
Participant Variables	Ages	Children (under 13), adolescents (13–18), Adults (above 18).
	Type of population	General population, clinical population
Treatment Variables	Type of writing	Free writing, structured writing
	Number of sessions	1–4 sessions, 5–8 sessions, over 9 sessions
	Length of 1 session	15 or fewer minutes, above 15 min
Measurement variables	Measuring area (Dependent variable)	Psychological health, physical health, General functioning
	Timing of measuring	Post, follow-up

#### 2.3.3 Test of homogeneity

Prior to analysis, a homogeneity test was performed, and publication bias was checked. Homogeneity was evaluated using the Q, and the heterogeneity test was performed using *I*^2^. The studies to be analyzed were heterogeneous (*Q* = 455.65, df = 312, *p* < 0.0001). Excess variance *I*^2^was 31.5%. A random effect model was used to calculate the effect size and the difference in effect size according to subgroups was analyzed.

#### 2.3.4 Publication bias check

**FIGURE 2:**
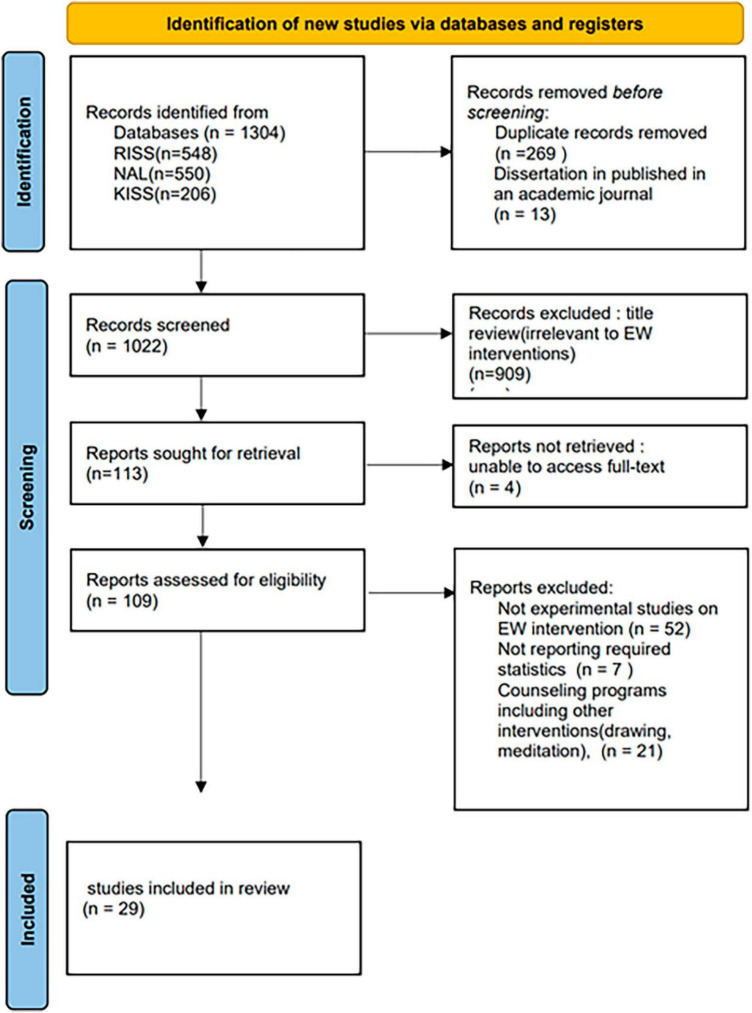
PRISMA flowchart for data collection.

**FIGURE 3:**
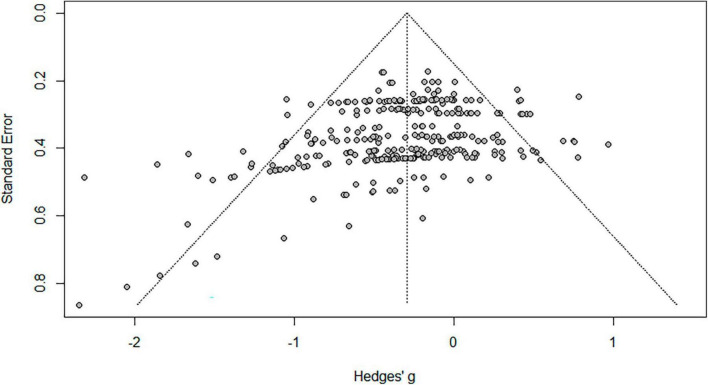
Correction of publication bias/.

Publication bias was analyzed using Funnel Plot and Trim-and-fill (73). The result of the funnel plot test was not symmetrical (Figures 2, 3). The result of Egger’s regression test through “egger.test()” of the Dmeter R package was statistically significant (*t* = −4.661, 95% CI −1.67–0.68, *p* < 0.001). Effect size was calculated using the Trim and Fill.

### 2.4 Effect size synthesis and a random effect model

Effect size was calculated using the “dmetar,” “meta,” and “metafor” packages of the statistical program R4.0.4. Since the effect size tends to be overestimated when the sample size is small, effect size was calculated using Hedges’ g in this analysis (77). We applied the Hedge’s g effect size calculation method presented by Borenstein et al. (78) The individual effect size along with 95% confidence intervals was calculated as follows.


$$g=d\left(1-\frac{3}{4df-1}\right),\;d=\frac{\bar{D_{T}}-\bar{D_{C}}}{{\text{s}}_{p}},$$

$${\text{s}}_{p}=\sqrt{\frac{\left(n_{1}-1\right)S_{T}^{2}+\left(n_{2}-1\right)S_{C}^{2}}{\left(n_{1}+n_{2}-2\right)}}$$

Note. *n*_*C*_ sample size of control group, *n_t_* = sample size of treatment group, *s*_*p*_ pooled standard deviation within treatment and control group, $\bar{D_{T}}$ = mean difference of pre- and post- score of treatment group, $\bar{D_{c}}$ = mean difference of pre-and post- score of control group.

Because the studies included in the meta-analysis were designed with more than one dependent variable, the effect size was more than one. Consequently, we aggregated effect size within each study and used all study level effect sizes to calculate the global effect size as suggested by Cooper (79).

## 3 Results

### 3.1 Study characteristics

A total of 29 studies were selected for final analysis. Table 2 shows the descriptive characteristics of these studies.

**TABLE 2:** Descriptive characteristics of the studies.

Study	*N*	Ages	*P*	TW	NS	LS	TM	DV
1	E: 15 C: 15	B	Cli	F	3	30	Fo	Psy, Phy, Func
2	E: 8 C: 7	A	Cli	S	3	20	Po, Fo	Psy
3	E: 11 C: 11	C	Cli	F	3	20	Po	Psy
4	E: 31 C: 29	C	Cli	F	1	NR	Po	Psy
5	E: 15 C: 15	A	Cli	F	3	30	Po, Fo	Psy, func
6	E: 11 C: 11	C	Cli	F	5	25	Po, Fo	Psy, func
7	E: 38 C: 42	B	Gen	S	6	25	Po	Psy
8	E: 23 C: 23	B	Cli	S	3	NR	Po, Fo	Psy
9	E: 13 C: 11	C	Gen	F	3	20	Po, Fo	Psy, Phy, Func
10	E: 23 C: 23	C	Cli	F	4	15	Po, Fo	Psy, Phy
11	E: 15 C: 15	A	Cli	F	3	30	Po, Fo	Psy, Phy, Func
12	E: 18 C: 18	C	Cli	F	1	30	Po, Fo	Psy, Phy, Func
13	E: 15 C: 15	A	Cli	S	3	20	Po, Fo	Psy
14	E: 28 C: 28	C	Gen	F	14	NR	Po	Psy
15	E: 16 C: 16	C	Cli	S	4	30	Po	Psy, Func
16	E: 46 C: 17	C	Cli	F	3	10	Po, Fo	Psy,
17	E: 6 C: 5	C	Cli	S	4	20	Po, Fo	Psy, Func
18	E: 31 C: 30	B	Gen	S	14	50	Po, Fo	Psy,
19	E: 11 C: 14	C	Gen	F	3	20	Po, Fo	Psy, Phy, Func
20	E: 30 C: 30	C	Cli	S	3	15	Po	Psy
21	E: 16 C: 15	C	Cli	S	4	20	Po, Fo	Psy
22	E: 12 C: 12	C	Cli	F	4	20	Po	Psy
23	E: 10 C: 7	C	Cli	S	4	20	Po, Fo	Psy
24	E: 12 C: 13	C	Gen	S	8	20	Po, Fo	Psy
25	E: 48 C: 48	C	Gen	S	6	20	Po, Fo	Psy, Func
26	E: 26 C: 24	C	Cli	F	3	30	Po	Psy
27	E: 64 C: 71	B	Gen	F	3	30	Po	Psy, Phy
28	E: 18 C: 16	C	Cli	S	4	20	Po	Psy
29	E: 30 C: 31	C	Gen	S	3	30	Po, Fo	Psy, Func

Age (A: under 13, B: 13–19, C: above 19), N: number of participants (E: experiment group, C: Control group), P: the type of population (Cli: Clinical population, Gen: General population); TW, type of writing (F: Free writing, S: structured writing); NS, number of sessions; LS, length of sessions in minutes (NR: Not reported); TM, timing of measuring (po: posttest, Fo: Follow-up test) post (1) follow-up (2); DV, dependent variable (Psy: psychological health, Phy: physical health, Func: general functioning).

### 3.2 Global effect size

**FIGURE 4:**
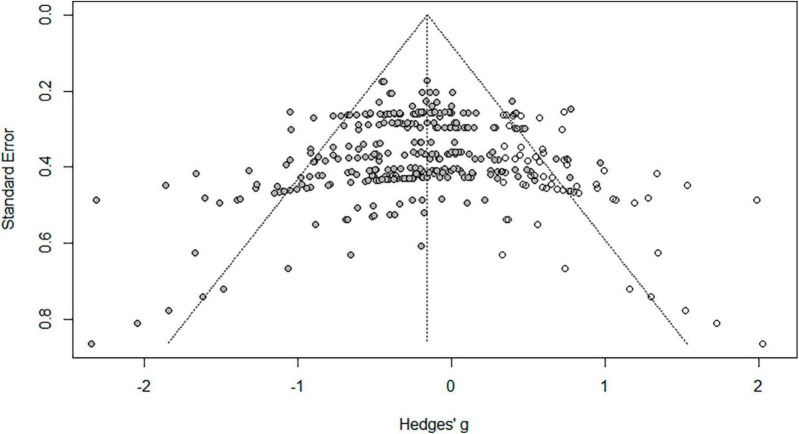
Forest test.

The overall effect size was *g* = 0.33 (*p* < 0.01), which indicates a medium effect (0.2 < *g* < 0.8) and its 95% confidence interval was situated between 0.22 and 0.44. The forest plot (Figure 4) shows the effect size of each article. Because the funnel plot test is not symmetrical, the effect size was calculated using the Trim and Fill. A total of 62 correction values were used and the effect size after the correction was 0.16 (*P* < 0.001).

### 3.3 Moderating variables

#### 3.3.1 Variables of participants

Table 3 shows the result of participants variables. First, the effect size was larger in the adolescents’ group (*g* = 0.311) than in the adults’ group (*g* = 0.29), and larger in the children’s group (*g* = 0.319) than in the adolescents’ group. However, this was not statistically significant. Second, the effect size was larger in the clinical population (*g* = 0.308) than in the general group (*g* = 0.272) without complaint problems. However, this was also not statistically significant.

**TABLE 3:** Effect size by participants’ characteristics.

		*K*	ES (g)	SE	*P*	95%		Test for subgroup difference		
						**LL**	**UL**	***Q***	**df**	***p***
Ages	Children (under 13)	44	0.319	0.097	<0.001	0.509	0.129	0.142	2	0.931
	Adolescent (13–18)	27	0.311	0.077	<0.001	0.463	0.159			
	Adults (above 18)	242	0.290	0.026	<0.001	0.341	0.239			
Type of population	General population	83	0.272	0.041	<0.001	0.353	0.192	0.491	1	0.483
	Clinical population	230	0.308	0.030	<0.001	0.368	0.248			

K, the number of effect sizes; g, Hedges’ g; SE, standard error; LL, confidence intervals lower limit; UL, confidence intervals upper limit.

#### 3.3.2 Treatment variables

Table 4 shows the result of treatment variables. The effect size was larger in the structured writing (*g* = 0.385) than in the free writing (*g* = 0.198) group (*p* < 0.001). Second, the effect size was larger in 5–8 sessions (*g* = 0.373) than in 1–4 sessions (*g* = 0.265), and larger in 9 sessions (*g* = 0.599) than in 5–8 sessions (*p* < 0.05). Third, the effect size was larger in the case of more than 15 min (*g* = 0.351) than in the case of 15 min or less (*g* = 0.101) per session (*p* < 0.001).

**TABLE 4:** Effect size by research design.

		*K*	ES (g)	SE	*P*	95%		Test for subgroup difference		
						**LL**	**UL**	***Q***	**df**	***p***
Type of writing	Free writing	148	0.189	0.035	<0.001	0.259	0.12	16.286	1	<0.001
	Structured writing	165	0.385	0.033	<0.001	0.451	0.32			
Number of sessions	1–4 sessions	235	0.265	0.029	<0.001	0.321	0.209	8.171	2	<0.05
	5–8 sessions	73	0.373	0.052	<0.001	0.475	0.271			
	Over 9 sessions	5	0.599	0.139	<0.001	0.871	0.328			
Length of 1 session	15 or less minute	47	0.101	0.039	<0.001	0.177	0.024	37.608		
	Above 15 min	260	0.351	0.029	<0.001	0.407	0.294		2	<0.001
	Not reported	6	0.120	0.036	<0.001	0.190	0.051			

K, the number of effect sizes; g, Hedges’ g; SE, standard error; LL, confidence intervals lower limit; UL, confidence intervals upper limit.

#### 3.3.3 Measurement variables

Table 5 shows the result of measurement variables. First, analyzing the difference in the effect size according to the category of the dependent variable, general functioning (*g* = 0.317) was the largest, followed by psychological health (*g* = 0.297), and then reported physical health (*g* = 0.242). However, this was not statistically significant. Second, in case of the timing of the test, the follow-up (*g* = 0.366) showed a larger effect size than the post-test (*g* = 0.258).

**TABLE 5:** Effect size by measurement.

		*K*	ES (g)	SE	*P*	95%		Test for subgroup difference		
						**LL**	**UL**	***Q***	**df**	***p***
Measuring area (Dependent variable)	Psychological health	238	0.297	0.027	<0.001	0.350	0.244	0.483	2	0.78
	Reported physical health	18	0.242	0.084	<0.001	0.409	0.077			
	General functioning	57	0.317	0.072	<0.001	0.460	0.175			
Timing of test	Post	197	0.258	0.031	<0.001	0.318	0.198	4.3440	1	<0.05
	Follow-up	116	0.366	0.042	<0.001	0.448	0.284			

K, the number of effect sizes; g, Hedges’ g; SE, standard error; LL, confidence intervals lower limit; UL, confidence intervals upper limit.

## 4 Discussion

To examine the effects of EW intervention conducted in Korea, this study sought to find the overall average effect size of EW interventions in studies published until 2021 and identify moderate variables. First, the overall average effect size revealed through this study was 0.33, which is the median effect size. When publication bias was corrected, the effect size was 0.16, which means that the EW interventions benefit Koreans. Although the effect size is not large, it means that EW intervention is at least harmless and has a positive effect considering the efficiency and conciseness of interventions.

The effect size of 0.16 obtained in this study is as suggested by Frattaroli’s effect size of 0.15 (2). In other words, EW intervention was neither more effective nor less harmful to Asians than to individuals in Western cultures. This is in line with the results of Frattaroli’s study (2), which did not find a difference in effect according to race. This suggests the potential lack of difference in the EW intervention according to emotional suppression. However, it should be interpreted with caution. Emotional suppression and ethnicity are closely related but independent variables. In interpreting the results, it is necessary to consider that the psychological conflict behind emotional suppression may have a greater effect on psychological health (80, 81).

Another factor to consider is whether there is sufficient emotional exposure during EW interventions. Lu and Stanton argued that writing intervention could be more effective for Asians because emotional suppression could effectively reduce the conflict between the desire to express emotions and social and environmental constraints that prevent such expressions (56). However, the effect of emotional disclosure did not appear sufficient because the intervention itself was unfamiliar and awkward to Asians. Niles et al. suggested that the manipulation of EW interventions should include pre-practice so that individuals with low expressiveness would feel more comfortable before the actual intervention (82).

Another condition to be considered in relation to the degree of deep exposure is the presence of an audience, which may be a moderator for psychological health outcomes. In Frattaroli’s meta-analysis (2), studies in which participants’ disclosure was private had larger effect sizes than studies in which participants’ disclosure was turned into the experimenter. This can be a more important variable in Asian cultures, where individuals place great importance on their faces—such as for expressing dignity and honor. Therefore, the difference in the degree of exposure depends on whether the writing is submitted to the experimenter or not, and there is a possibility that the effect may be different. In other words, if there is a condition that the writing has to be submitted, deep exposure might not be achieved. Unfortunately, most studies did not mention the form in which writing results are to be submitted, so it could not be confirmed as a major variable in this study.

Second, there was no difference in the effect size according to the age of the participants. This shows that EW interventions are effective at a similar level for all age groups, rather than being effective for a specific age group. In previous studies (2, 52), there was no significant difference in effect size according to age. However, a study meta-analyzed the effect of EW interventions for adolescents (83) between the ages of 10 and 18. In the study, the effect of EW interventions in the adolescent group is said to be between 0.107 and 0.246, which was slightly smaller than the result obtained in the adult group of 0.15–0.47. In this study, although the difference was not statistically significant, the effect size was slightly smaller as the age increased.

There was no difference in effect size according to the type of population. In other words, no significant difference was found in the effect of EW intervention in the general population without any complaints or in the clinical group experiencing complaints or specific difficulties. This result is in line with the results of Frattaroli (2). This suggests that EW interventions can be helpful even in the general population without apparent psychological distress or difficulties.

However, it does not mean that the general populations selected without separate screening are psychologically healthy. Because nobody is completely free from stress or difficulties, the general population may also be experiencing stress or difficulties at a mild level. For example, in the study by Choi (84), general college students were recruited for the EW intervention without screening, and the writing topic was “difficulty in interpersonal relationships.” Also, in the case of Kim and Shin (85), general high school participants were asked to write about “academic stress.” Therefore, the general population that experiences stress or psychological difficulties even at a slight level could benefit from the intervention.

In these studies, participants were divided into two groups: the general and clinical populations. However, even within the same clinical populations, the spectrum can be very diverse—from those with severe disabilities and difficulties to those with mild problems. A meta-analysis on only the clinical group was conducted (5). In this study, the effect size was 0.19, which was relatively low—there were also studies with reported negative effect sizes in Frisina’s meta-analysis (5). This suggests that EW interventions may not be helpful for individuals with very severe trauma or psychological disorders. In fact, looking at previous studies, we can see that the effect of EW interventions did not exist when the symptoms were severe, such as high levels of PTSD (22) and eating disorders (47). Pennebaker also revealed that the group in which EW intervention was most effective was the one in a mildly stressful environment (3). The effect of EW intervention may differ depending on the severity of participants’ symptoms. More research is needed to determine what kind of individuals get the benefit and its boundary conditions.

Third, structured writing, in which the topic and format of each session are provided, has a greater effect than free writing in which individuals freely write down their deep thoughts and feelings about events related to stress or trauma. Although both free writing and structured writing commonly include the process of self-opening, structured writing provides a clearer topic and structure than free writing, making the intention of treatment and manipulation of the content clearer (84, 86). This is in line with the results of previous studies in which the effect size was found to have increased when the writing topic was more specific (2, 87).

Time per session and the total number of sessions are moderators of EW intervention. Pennebaker’s initial model was set to 4 sessions and 15 min per session, which came from the practical issues of reserving the experimental site rather than having a specific theoretical background (88). Since then, related studies have been expanded. Pennebaker has also recommended giving sufficient time to write (19). In this study, the effect of the intervention increased when the time per session exceeded 15 min. In case of the number of sessions, the effect size was large in the interventions of four or more sessions. This is similar to the results of previous studies; the larger the number of sessions, the larger the effect size (2, 52). These results can be explained by the point that a short session or time only promotes negative emotions or thoughts but does not give sufficient time to cognitively work on them (89). However, studies have also shown positive effects with only a single session (37, 90). Further, in the case of studies in which several sessions were conducted but the effect was measured at the end of each session, the largest effective change reportedly occurred after the first session (41, 91). There may be a non-linear relationship among the number of sessions, the time per session, and the effect size. For example, in Kim et al.’s study (92) on the effect of a group program on emotional regulation, though it was not about EW interventions, the emotional expression score of the 1st to 3rd sessions was not statistically significant; however, after the 4th session, a statistically significant difference appeared, showing the largest difference in the 8th session. Therefore, additional research is needed to explore how effect size changes as the number of sessions or time per session increases.

At the time of measurement, the effect size of the follow-up test was larger than that of the post-test. This means that the benefits from writing interventions are greater afterward than immediately after writing. Interestingly, half of the studies (15 of 29) conducted the follow-up test within 1–2 weeks after interventions, which is a very short time compared to that in Western studies. In the meta-analysis of Frattaroli (2), the average follow-up time was approximately 3 months after interventions. Although the negative effects of EW interventions are assumed to wear off in 1–2 h (5, 52, 93), meta-analyses excluded studies with follow-up periods of less than 1 month because of concerns about the impact of the short term. In this meta-analysis, the longest follow-up time was 8 weeks after interventions (93), whereas a study measured the change since the disclosure as late as 15 months after the intervention (48). This is because studies on Korean psychological intervention mainly focus on measuring performance immediately after a session within a few weeks, and studies confirming continuous performance are rare (93). In the meta-analysis of Frattaroli (2), larger effect sizes were found less than a month after writing than after a month. Therefore, when the benefit appears and how long it lasts should be studied.

When considering the implications of these findings for designing EW interventions for Koreans, several key considerations come to the forefront. Firstly, measures should be taken to ensure that sufficient emotional exposure occurs during the writing process. This may involve incorporating a pre-practice stage to familiarize individuals with expressing emotions through writing, as it can be both an unfamiliar and potentially uncomfortable experience. Additionally, time per session and the total number of sessions are identified as significant moderating variables, providing sufficient time and sessions for meaningful exposure is crucial. Secondly, The effect of structured writing was significantly greater than that of free writing, it suggests the need for further development of diverse structured topics and prompts in EW interventions. This indicates a potential for enhancing interventions by offering a variety of structured themes and instructions. Lastly, it is imperative to consider the notable finding that the effect size of follow-up assessments was larger than that of post-tests. Given that Korean studies have tended to set relatively shorter follow-up periods compared to Western studies, there is a need to extend the tracking period to more accurately measure the lasting impact of interventions.

The limitations of this study and suggestions for follow-up studies are as follows. First, most of the studies included in the study used self-report measures. Since this study covers all studies published in Korea, even if the variables are coded with the same value, the content is quite broad and heterogeneous, so the analysis could not possibly detect subtle differences.

Second, the categories of variables were not biased in measuring benefit. In the case of Western studies, the benefit of EW interventions has been extensively demonstrated in physical, psychological, and adaptive dimensions. In a study comparing the benefits of EW interventions among races, Asians particularly benefit from reducing physical symptoms than Westerners. However, most of the studies conducted in Korea measured psychological factors such as depression and anxiety, and the proportion of studies using physical symptoms as a dependent variable was not high. The dependent variable biased toward one category may have influenced the study results.

Third, the positive/negative dependent variables should be separated for the benefit of writing interventions. A decrease in negative emotions such as depression and anxiety, through writing, and an increase in positive factors such as wellbeing in life, can be different. In this study, all variables were treated in the same direction through reverse coding, but the effect may be different. Therefore, in the follow-up study, it is necessary to examine whether there is a difference in the effect by dividing it into positive/negative domains.

## Data availability statement

The raw data supporting the conclusions of this article will be made available by the authors, without undue reservation.

## Author contributions

YL: writing of the first and final draft of the manuscript, acquisition of data, interpretation of analysis, concept and design of the research, and final approval for publication. DK: writing of the first and final draft of the manuscript, interpretation of analysis, and final approval for publication. J-EL: acquisition of data, interpretation of analysis, concept and design of the research, and final approval for publication. All authors contributed to the article and approved the submitted version.
